# Functional Elastic Hydrogel as Recyclable Membrane for the Adsorption and Degradation of Methylene Blue

**DOI:** 10.1371/journal.pone.0088802

**Published:** 2014-02-20

**Authors:** Song Bao, Dongbei Wu, Qigang Wang, Teng Su

**Affiliations:** 1 Department of Chemistry, Tongji University, Shanghai, China; 2 Advanced Research Institute, Tongji University, Shanghai, China; Brandeis University, United States of America

## Abstract

Developing the application of high-strength hydrogels has gained much attention in the fields of medical, pharmacy, and pollutant removal due to their versatility and stimulus-responsive properties. In this presentation, a high-strength freestanding elastic hydrogel membrane was constructed by clay nanosheets, N, N-dimethylacrylamide and 2-acrylamide-2-methylpropanesulfonic acid for adsorption of methylene blue and heavy metal ions. The maximum values of elongation and Young’s modulus for 0.5% AMPSNa hydrogel were 1901% and 949.4 kPa, respectively, much higher than those of traditional hydrogels. The adsorptions were confirmed to follow pseudo-second kinetic equation and Langmuir isotherm model fits the data well. The maximum adsorption capacity of hydrogel towards methylene blue was 434.8 mg g^−1^. The hydrogel also exhibited higher separation selectivity to Pb^2+^ than Cu^2+^. The methylene blue adsorbed onto the hydrogel membrane can be photocatalytically degraded by Fenton agent and the hydrogel membrane could be recycled at least five times without obvious loss in mechanical properties. In conclusion, this presentation demonstrates a convenient strategy to prepare tough and elastic clay nanocomposite hydrogel, which can not only be applied as recyclable membrane for the photocatalytic degradation of organic dye, but also for the recovery of valuables.

## Introduction

Hydrogels are three-dimensional hydrophilic polymers capable of absorbing and retaining water within the network without disintegrating [1], [2]. In recent years, polymer hydrogels have also generated great interests in the application areas such as pollutant removal, biosensors, artificial muscles, and drug delivery devices, due to their versatility and stimulus-responsive properties [3]–[5]. However, the use of hydrogels in several applications is limited since most of them are brittle, soft, and typically exhibit low mechanical strength and poor stability [6].

To solve this problem, intense efforts are devoted to synthesizing hydrogels with improved mechanical properties. As a pioneering example, clay nanocomposite hydrogel shows ultrahigh tensile strength and elongation due to the multiple non-covalent effects between mono-dispersed clay nanosheets and polyacrylamide chains [7]–[10]. Aida *et al.* has developed the supramolecular hydrogel by mixing sodium polyacrylate dispersed clay nanosheets with dendritic polymers *via* multiple salt bridge [11]. Nevertheless, no much information is available on the functionalization of high-strength hydrogel for practical applications.

Recently, our group reported that the dispersed semiconductor nanoparticles, such as TiO_2_, ZnO, CdS, *etc*., can initiate the polymerization of monomers under sunlight and crosslink themselves to form tough nanocomposite hydrogels with the aid of clay [12]. These clay nanocomposite hydrogels exhibit excellent photocatalytic properties. As a continuous work, herein clay nanocomposite hydrogels were further functionalized and applied for adsorption of organic dyes such as methylene blue (MB), and heavy metal ions, the removal of which has drawn increasing attention in recent years due to their long-term environmental toxicity and short-term public health damage. Here, a self-standing membrane, produced from tough and elastic hydrogel, was used for the adsorption study, instead of shapes from other groups, where fragile hydrogel particles or dried gel powders are commonly employed [13]–[15]. 2-acrylamide-2-methylpropanesulfonic acid sodium salt (AMPSNa) was chosen as a modifier and added into the hydrogel network to improve adsorption efficiency, since –SO_3_H groups in the AMPSNa have a high affinity towards dye molecules and heavy metal ions. In brief, this membrane is characterized by easy operability, low possibility of introducing second-hand pollution from adsorbents, high adsorption capacity and reusability, which is a kind of environment-friendly separation technology in biological cell sorting, industrial effluent detoxification and valuables recovery [16], [17].

## Materials and Methods

### Materials

N, N-dimethylacrylamide (DMAA ≥99.0%) and 2, 2-diethoxyacetophenone (DEAP, 98%) were purchased from TCI Shanghai. Clay nanosheets (Clay-NS, Laponite XLG, Rockwood Ltd., molecular formula of Mg_5.34_Li_0.66_Si_8_O_20_(OH)_4_), 2-acrylamide-2-methylpropanesulfonic acid (AMPS, 98%, Alfar Aesar) and methylene blue trihydrate (MB ≥98.5%, Shanghai Qiangshun Chemical Reagent Co., Ltd.) were used as received. Acid fuchsin (BS), crystal violet (AR), Cu(NO_3_)_2_·3H_2_O (AR), Pb(NO_3_)_2_ (AR), NaOH (AR), NaCl (AR), H_2_O_2_ (30%), and (NH_4_)_2_Fe(SO_4_)_2_·6H_2_O (AR) were obtained from Sinopharm Chemical Reagent Co., Ltd. The methylene blue (MB) is chosen as a model dye.

### Clay Nanocomposite Hydrogel Preparation

Typically, DMAA (0.232 mL) with 2, 2-diethoxyacetophenone (photoinitiator, 0.5% relative to monomer) and clay-NS (0.25 g) were successively dissolved in distilled water (4.025 mL) to produce a transparent clay dispersed solution at room temperature. After 5-minute stirring, 0.5 mL of AMPSNa solution with the weight concentration of 5.0% was added to the as-dispersed solution, which made the mixture sticky and hard to stir due to the strong electric interaction between clay-NS and AMPSNa. Then a centrifugal agitator (HS1000, Dongguan Bangangde Electronics Co., Ltd.) was used for smoothing surface of the mixture. The final transparent and uniform poly(DMAA-co-AMPSNa)/clay nanocomposite hydrogel could be prepared by UV light irradiation for 1 h (average 22.4 mw cm^−2^ at 365 nm, Maxima ML-3500C/F, Spectronics Corp, USA). In the whole experiment, the mass fraction of monomer, clay-NS and water was kept to be 5%, 5% and 90%, respectively, otherwise specific statement.

### Swelling Measurement

Disc-shaped hydrogels were soaked in excessive distilled water or 0.9% NaCl solution at room temperature for 3 days until swelling equilibrium was reached. The swelling ratio, *S_R_*, was obtained gravimetrically, which can be calculated as follows:
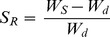
(1)where *W_S_* and *W_d_* are the mass of swollen and dry sample, respectively. The reported results were averaged on five independent runs.

### Adsorption Research

#### Membrane adsorption

**Figure 1:**
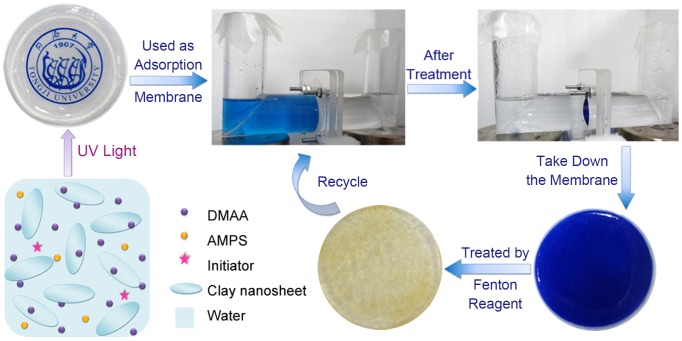
Proposed scheme for recyclable MB treatment and optical images of the hydrogel membrane.

Before membrane adsorption, the as-prepared fresh hydrogel was immersed into 0.9% sodium chloride solution to get a swelling equilibrium, and then flattened by a tensile-compressive tester (FR-108B, Rario Co.) to produce an elastic hydrogel membrane with the diameter of 5 cm and thickness of 2.5 mm. After immobilization of the hydrogel membrane onto a special instrument as shown in Figure 1, adsorption experiments were carried out at room temperature (approx. 25°C) with magnetic stirring (200 rpm). 150 mL of MB solution was poured into the left organic glass vessel. The initial concentration of MB ranges from 10.0 mg L^−1^ to 162.6 mg L^−1^ at a constant pH of 6.5. An equal volume of 0.9% NaCl solution was added into the right container in order to balance osmolality, maintain constant ionic strength, mimic actual industrial wastewater environment, and avoid excessive swelling of the hydrogel membrane. After a period of time, MB could be completely adsorbed by the membrane. The residual solution appeared almost transparent and was drawn for analysis by UV-vis spectrophotometry (UV-2700, Shimadzu) at the maximum absorbance of 664 nm. All experiments were triplicated.

### Hydrogel Resin or Powder Batch Adsorption

Hydrogel resin or powder batch adsorption was carried out by suspending 30 mg dry hydrogel resin (diameter around 1 mm) or lyophilized hydrogel powder (diameter around 40 µm) in 100 mL of MB solution at a certain concentration. Suspensions were shaken by an oscillator (TQZ-312, Shanghai Jing Hong Laboratory Instrument Co., Ltd.) at 25°C and 200 rpm. The contacting time is 4 h for resin and 30 min for powder, respectively. After that, the mixture was centrifuged and the residual MB concentration in the solution was analyzed. The equilibrium adsorption capacity, *Q_e_* (mg g^−1^), is the amount adsorbed per gram of dry gel at equilibrium, which can be calculated by the following equation:
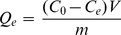
(2)where *C_0_* and *C_e_* are the initial and equilibrium concentration of MB in the solution (mg L^−1^), respectively, *V* is the volume of the solution (L), and *m* is the weight of the dry hydrogel used (g).

*R_t_* (%) is defined as the removal ratio of MB at specific time *t* (h), and is calculated as follows:
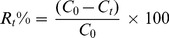
(3)where *C_t_* (mg L^−1^) is the concentration of MB at specific time.

### Photocatalytic Degradation of MB

For photocatalytic degradation of methylene blue, the initial concentration of MB of 10 mg L^−1^ was chosen for adsorption. After nearly entire uptake of methylene blue onto the hydrogel membrane, the membrane was taken down from the special instrument and immersed into a solution containing Fenton reagent, the composition of which is 8 mL of H_2_O_2_ (30%) and 72 mL of Mohr’ salt (FeSO_4_(NH_4_)_2_SO_4_·6H_2_O, 10 mM). 10 minutes later, the adsorbed MB was photo-catalytically degraded under UV irradiation (Maxima ML-3500C/F, Spectronics Corp, USA) and the hydrogel membrane was refreshed. Such adsorption and photocatalytic degradation cycle was repeated at least five times, and nearly 100% adsorption and photocatalytic degradation can be achieved in each cycle.

### Characterizations

For mechanical measurements, a tensile-compressive tester (FR-108B, Farui Co.) was used for compressive stress–strain measurements. The gel sample was prepared in cylindrical shape with the diameter of 13.0 mm and gauge length of 13.2±0.2 mm. 95% compression was performed at a compressive rate of 1.0 mm min^−1^. Tensile properties were measured by the same machine using a dumbbell-shaped sample (20 mm×2 mm×2 mm) at a stretching rate of 10.0 mm min^−1^. The Young’s modulus of these two kinds of mechanical properties is determined according to the strain range from 5% to 15%. The results were averaged on at least three independent runs.

A scanning electron microscopy (SEM, Hitachi S-4800, JEOL) was employed for observing the microstructures of the hydrogel. TEM images of bio-cutting samples were obtained by a transmission electron microscope (TEM, JEM-2010, JEOL) at an accelerating voltage of 120 kV.

## Results and Discussion

### Morphology of Hydrogel Membrane

**Figure 2:**
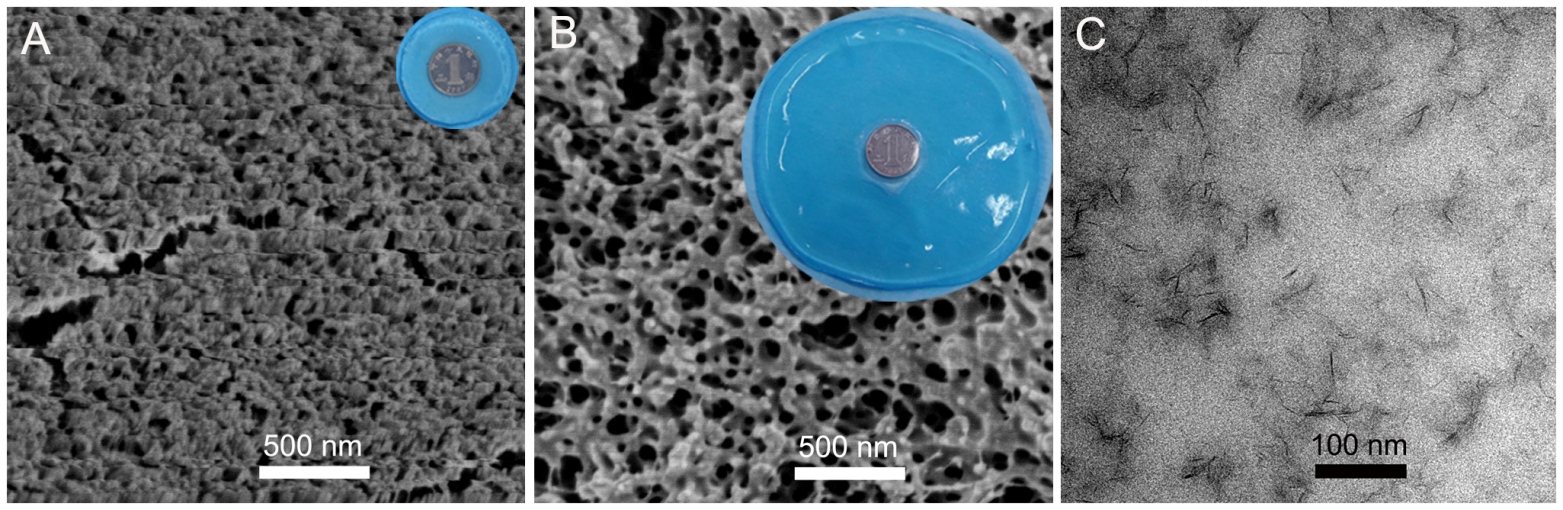
SEM and TEM images of hydrogel with the AMPSNa concentration of 0.5%. (A) SEM image of the fresh hydrogel and its photo at the top-right corner. (B) SEM image of the hydrogel swollen in distilled water and its photo at the top-right corner. (C) TEM image of fresh hydrogel.

Figure 2A and Figure 2B show SEM images of hydrogel with 0.5% AMPSNa before and after swelling in distilled water. For the fresh one, the surface of hydrogel is rough and uneven with irregular small pores. While for the swollen hydrogel, relative regular pores with sizes ranging from several tens to several hundred of nanometer can be observed. Looser pore structure of the hydrogel after swelling than the fresh one suggests rapid adsorption kinetics. TEM image reveals that the mono-dispersed clay-NS exist in the gel (Figure 2C), which have the same size as in solution. Here, the mono-dispersed clay nanosheets would act as crosslinkers in the hydrogel, leading to excellent mechanical and swelling properties.

### Swelling Properties of Hydrogel

Since ionic strength has an obvious effect on the swelling ratio, the swelling tests were performed in distilled water and 0.9% NaCl solution, respectively (Table S1). With increasing concentrations of AMPSNa, the swelling ratio in distilled water increases and the maximum value can reach 225.1±5.2 at the content of AMPSNa of 0.7%, much higher than values obtained from Haraguchi [7]. It is evident that the strong association of AMPS will produce a repulsive force between the negative-charged groups in AMPS, which makes the polymer chains more stretchable and then benefits the penetration of water molecules into the hydrogel networks, thus resulting in improved swelling capacities. Furthermore, the polymer chains in the poly(DMAA-co-AMPSNa) hydrogels can strongly absorb more water than the DMAA hydrogel. For 0.9% NaCl solution system, the swelling ratio maintains around 17, which might be due to the weak electrostatic repulsive force between AMPS groups at high ionic strength environment. Thus, in the following adsorption experiments, 0.9% NaCl solution was used to avoid excessive swelling and maintain the shape of hydrogel membrane.

### Mechanical Properties of Hydrogel

In preliminary experiments, we found if the concentration of clay-NS was less than 3%, the clay nanocomposite hydrogel would demonstrate weak mechanical properties. While the concentration of clay-NS was more than 5%, the clay-NS tended to aggregate. Moreover, it was observed that with increasing amounts of clay-NS, the mechanical strength of clay nanocomposite hydrogel would be enhanced obviously. Therefore, in follow-up experiments, the concentration of clay-NS of 5% was employed to construct a hydrogel of considerable mechanical strength. Detailed illustrations about the effect of clay-NS concentration on the mechanical properties of hydrogel can be found in earlier work [12].

**Figure 3:**
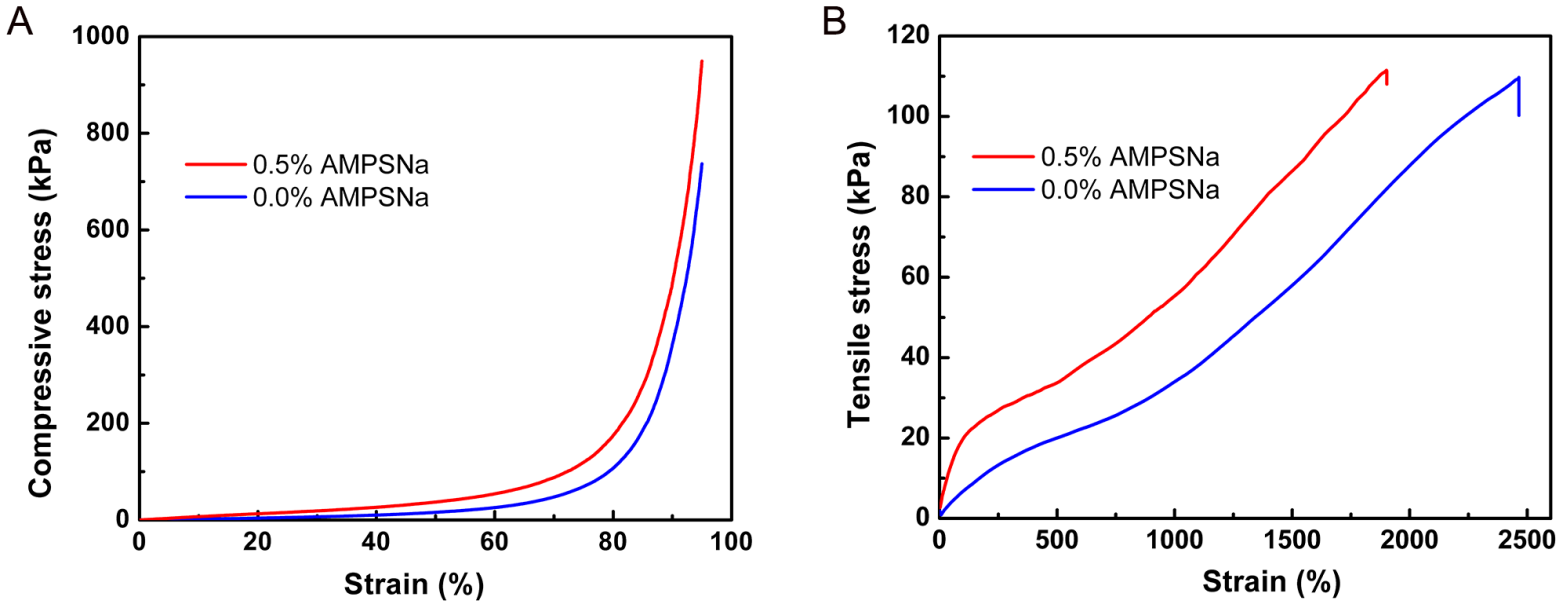
Mechanical properties of fresh hydrogels in various compositions. (A) Compressive properties. (B) Tensile properties.

Mechanical behaviors of hydrogels with various proportions or status were studied in detail and the results are listed in Table S2. The mechanical properties (compressive and tensile performances such as modulus, strength and elongation) strongly depend on the content of AMPSNa and hydrogel status. Young’s modulus can be calculated from the linear part of stress (kPa) versus strain (%) plots (strain range from 5% to 15%). For the compressive tests, we found that all hydrogels can tolerate a strain greater than 95% and exhibit partially rubber-like properties both in fresh status and in 0.9% NaCl solution. The values of Young’s modulus and compressive strength increase with increasing AMPSNa content in the range of 0–0.5%, and then decrease with further increasing AMPSNa amounts from 0.5% to 0.9% for both fresh and swollen hydrogels. The maximum Young’s modulus and compressive strength for the fresh hydrogel whose composition is 0.5% AMPSNa, 4.5% DMAA, 5% Clay-NS and 90% water are 64.9 kPa and 949.4 kPa, respectively. As shown in Figure 3A, the compressive stress for 0.5% AMPSNa fresh hydrogel is higher than that for 0.0% AMPSNa fresh hydrogel over the entire range of strain. A reasonable explanation for the enhancement of compressive strength is that there exist more dispersed polymer chains carrying their electrostatic exclusive force in the hydrogel. Figure S1A presents the effect of hydrogel status on the compressive stress with various AMPSNa concentrations. It can be found that the fresh hydrogel has the largest compressive stress, hydrogel swelled in 0.9% NaCl solution in the middle, and hydrogel swelled in distilled water at the weakest, on condition that three types of hydrogel have the same AMPSNa concentration. Even for 0.5% AMPSNa hydrogel swollen in distilled water, its compressive strength can achieve 246.2 kPa. So excellent mechanical properties suggest that clay nanocomposite hydrogel can not only be distorted to all kinds of shapes, but also resist the erosion of sewage during the process of wastewater treatment.

Figure 3B gives the plots of tensile stress (kPa) versus strain (%) for the fresh hydrogel whose concentration of AMPSNa is 0.5% and 0.0%, respectively. For the 0.5% AMPSNa hydrogel, the tensile modulus and strength are 29.4 kPa and 111.8 kPa, respectively, exhibiting a moderate mechanical character. The tensile modulus and strength of 0.5% AMPSNa hydrogel are higher than those of 0.0% AMPSNa hydrogel, but the maximum values of elongation for 0.5% AMPSNa hydrogel (1901%) is less than that for 0.0% AMPSNa hydrogel (2464%). The more rigid polymer chains with exclusive groups should determine the higher tensile strength and worse elongation. Similar tensile behaviors for 0.5% and 0.0% AMPSNa hydrogels in 0.9% NaCl solution can be observed in Figure S1B. It is easy to understand that the higher swelling ratio makes the gel network much looser, leading to a decrease in tensile property. Therefore, the tensile tests for hydrogel in distilled water couldn’t be performed due to their weak tensile strength. The maximum values of elongation for 0.0% and 0.5% AMPSNa hydrogel swelled in 0.9% NaCl solution are 1305% and 1286%, respectively, exhibiting a comparable tensile performance.

### Adsorption Research

**Figure 4:**
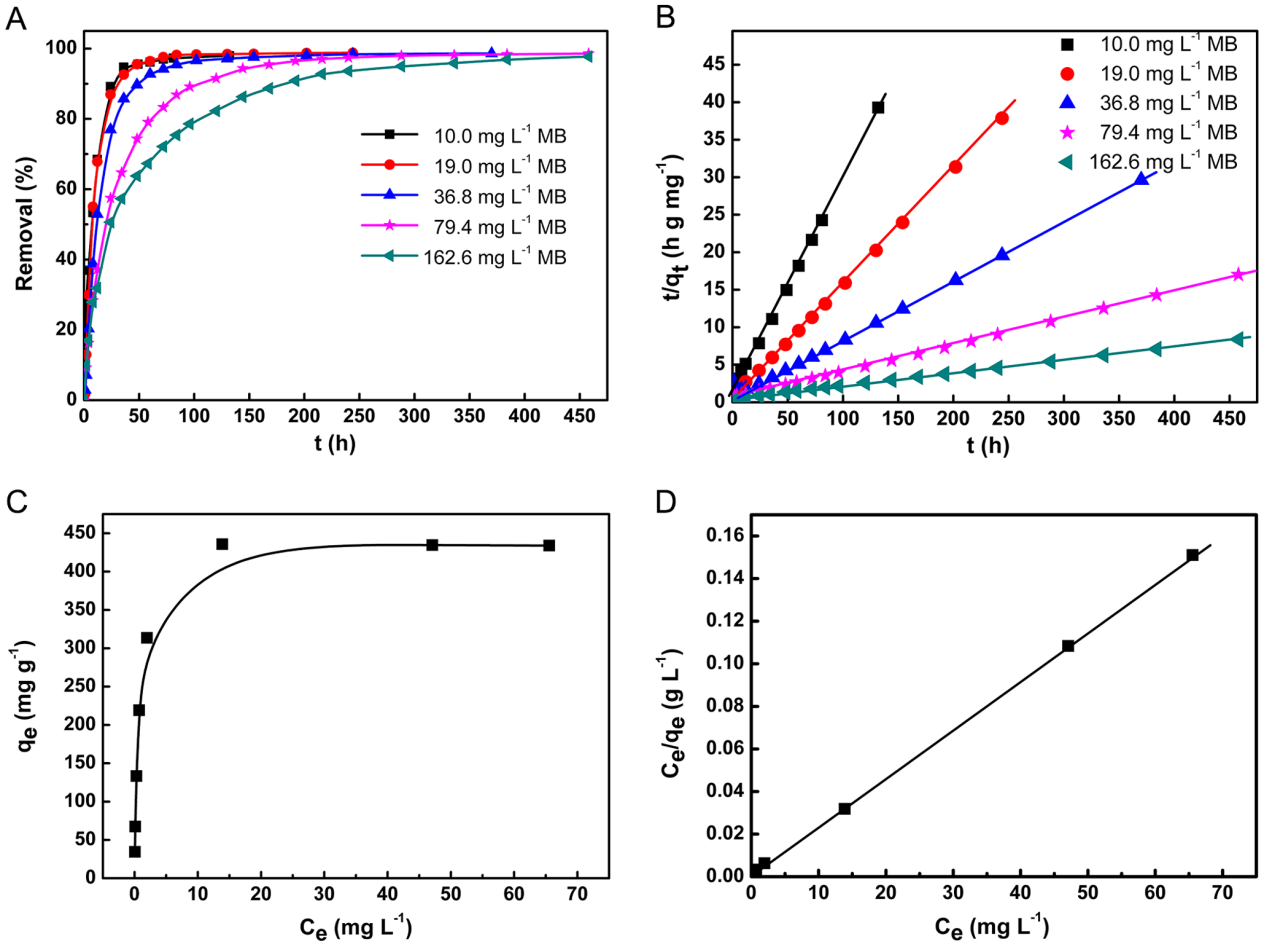
Adsorption performances of hydrogel containing 0.5% AMPSNa. (A) Time effect on the removal of methylene blue with various concentrations. (B) Pseudo-second-equation fitting for adsorption kinetics. (C) The effect of concentration of MB on the adsorption. (D) Langmuir equation fitting for adsorption isotherm. The volume of adsorbate *V* = 150 mL, the mass of fresh hydrogel membrane *m* = 5.0 g (0.436 g dry hydrogel), the temperature of adsorption *T* = 298 K, ionic strength was maintained by 0.9% NaCl solution, pH = 6.5.

As a typical application, the adsorption of MB onto hydrogel membrane was investigated under different conditions, including diverse initial concentrations of dyes and contact time. The contact time is one of the important parameters in adsorption process. If it is not enough to attain equilibrium, only a partial removal of solute can be achieved. Additionally, the rapid adsorption and high capacity are desirable properties for a sorbent. To study these properties, MB solution with different concentrations were added into the vessel and their removals were determined at time intervals. Changes in removal of the samples with time are given in Figure 4A. It can be observed that with increasing MB concentrations, more equilibrium time is required. When the initial concentration of MB is less than 20.0 mg L^−1^, nearly 100% removal can be achieved within 50 h. When the concentration of MB increases to 162.6 mg L^−1^, 100% removal needs 450 h. As usual, the equilibrium time is related to the specific surface area of sorbent. The larger the specific surface area, the shorter the equilibrium time of adsorption will be. Therefore, the adsorption tests with dry hydrogel resins (diameter around 1 mm) and hydrogel powder (diameter around 40 µm) were performed for comparison purpose. As expected, the entire removal of MB under the same condition can be finished in 4 h by resins and 30 min by powder, showing rapid adsorption kinetics. In addition, if we reduce the diameter or the thickness of the hydrogel membrane, the equilibrium time shortens correspondingly.

Both pseudo-first-order and pseudo-second-order equations were used for analyzing kinetic data. High correlation coefficients (*R^2^*>0.99) for all of MB with different concentrations show the pseudo-second-order equation fit the data well (Figure 4B). The differential form of a pseudo-second-order equation is expressed as the equation 4 [18]:
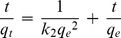
(4)


From Table 1, it can be observed that the equilibrium adsorption capacity increases with increasing initial concentrations of MB, which is attributed to adequate available binding sites from the hydrogel membrane. The adsorption rate is related to the concentration of active sites on the surface of adsorbent. Therefore, it can be deduced that for the same hydrogel membrane, the adsorption rate decreases when MB concentration increases. This is due to the competitive adsorption among MB molecules, which is confirmed by the values of rate constants from different MB concentrations (Table 1). The MB adsorption onto the hydrogel membrane could be a rate controlling process, which may involve valence forces through the associated electrons between dye and adsorbent [19]–[21].

**Table 1:** Kinetic parameters in the adsorption of methylene blue by clay nanocomposite hydrogel membrane (RT, pH of 6.5 and 150 mL).

Concentration of MB (mg L^−1^)	Experimental q_e_ (mg g^−1^)	*k_2_*×10^3^ (g mg^−1 ^h^−1^)	*R^2^*
10.0	3.36	54.67	0.9988
19.0	6.44	24.56	0.9985
36.8	12.52	5.60	0.9943
79.4	27.02	1.94	0.9996
162.6	55.06	0.65	0.9993

The effect of concentrations of MB on adsorption was performed at 298 K and the results are shown in Figure 4C. The adsorption capacity increases with the increase of equilibrium concentrations of MB and reached a constant value in high concentration range. Nearly 100% uptake of MB molecules onto the hydrogel membrane could be achieved with the equilibrium concentration of MB less than 13.9 mg L^−1^, and the initial concentration equals to 35.0 mg L^−1^. The Langmuir and Freundlich models were adopted for describing adsorption behaviors. They can be expressed as the equation 5 [22]–[24] and 6 [25], respectively, and shown as follows:
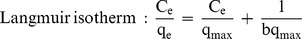
(5)




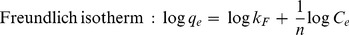
(6)where *q_max_* (mg g^−1^) is the maximum amount of adsorbate, and *b* (L mg^−1^) is the Langmuir parameter expressing an affinity of sorbate for the binding sites. *q_max_* and *b* can be determined from the linear plot of *C_e_/q_e_* versus *C_e_*. *K_F_* (L g^−1^) and *n* (dimensionless) are the Freundlich adsorption isotherm constants, indicative of the extent of adsorption and the degree of nonlinearity, respectively. The plot of ln*C_e_* versus ln*q_e_* for the adsorption was employed to generate the intercept value of *K_F_* and the slope value of *n*, respectively.

It is well known that Langmuir isotherm model represents monolayer adsorption occurring on an energetically uniform surface, where the adsorbed molecules are not interactive. Accordingly, equilibrium is attained once the monolayer is completely saturated. Contrary to Langmuir, Freundlich model describes the adsorption on an energetically heterogeneous surface, where the adsorbed molecules are interactive. All isotherm model parameters for the adsorption of MB are summarized in Table 2. It is evident from these data that the adsorption is fitted well to Langmuir isotherm model as indicated by the numerical values of the correlation coefficients (*R^2^*) (Figure 4D). A reasonable explanation is that MB molecules are monolayer-adsorbed onto the hydrogel surface *via* AMPSNa units on the polymer structure, since AMPSNa has the ability to create interactions with the amino groups of dye. There may also exist hydrophobic interactions between the aromatic rings of MB and the hydrophobic groups on the polymer [26], [27]. In this step, there is no interaction among MB molecules and the adsorption should follow Langmuir isotherm model.

**Table 2:** Langmuir and Freundlich isotherm constants (RT, pH of 6.5 and 150 mL).

Adsorptionisotherm	Langmuir			Freundlich		
	*q_max_*(mg g^−1^)	*b*(L mg^−1^)	*R^2^*	*k_F_*(L g^−1^)	*n*	*R^2^*
Isotherm constants	434.8	1.53	0.9999	154.8	3.05	0.8280

The maximum adsorption capacity of adsorbent calculated from Langmuir isotherm equation defines the total capacity of the adsorbent for MB molecules. It was calculated that the maximum adsorption capacity of hydrogel towards MB is 434.8 mg g^−1^, comparable with those of graphene oxide-chitosan hydrogels (390 mg g^−1^) [28] and graphene oxide sponge (387 mg g^−1^) [29], and much larger than those of chitosan beads (99 mg g^−1^) [30], C_2_-symmetric benzene-based hydrogels (53.38 mg g^−1^) [31] and swede rape straw (246.4 mg g^−1^) [32]. Although most of polymer adsorbents, such as sodium humate/polyacrylamide/clay hybrid hydrogels (800 mg g^−1^) [33], lignocellulose-*g*-poly(acrylic acid)/montmorillonite hydrogels (1994.38 mg g^−1^) [34] and poly(2-acrylamido-2-methylpropane sulfonic acid-co-itaconic acid) hydrogels (1000 mg g^−1^) [35], exhibit quite high adsorption capacities, they can not be regenerated and do not satisfy environmental requirements. Designing and synthesizing new types of hydrogel with efficient adsorption and regenerative properties is an essential subject in the removal of dyes from wastewater. Besides methylene blue, other dyes such as acid fuchsin and crystal violet were also employed for exploring adsorption characters of the clay nanocomposite hydrogel. As expected, the hydrogel exhibits quite high adsorption capacities. The maximum adsorption capacities towards acid fuchsin and crystal violet were calculated to be 416.7 mg g^−1^ and 232.0 mg g^−1^, respectively, according to Langmuir isotherm equation. Their illustrations of adsorption isotherm are given in Figure S2.

In order to explore adsorption performances of high-strength clay nanocomposite hydrogel towards heavy metal ions, Cu^2+^ and Pb^2+^ were chosen for investigations and the results are presented in Figure S3. The maximum adsorption capacities of Cu^2+^ (16.8 mg g^−1^) and Pb^2+^ (152.9 mg g^−1^) can be obtained, exhibiting excellent adsorption selectivity. The reason for hydrogel’s higher affinity towards Pb^2+^ than Cu^2+^ might be due to the stronger coordination ability of –SO_3_H with Pb^2+^ than Cu^2+^ [36]. More detailed studies on the adsorption of metal ions with high-strength clay nanocomposite hydrogel are under investigation.

Moreover, the permeability of the hydrogel membrane was concerned. 150 mL of 0.9% NaCl solution was poured into the left vessel to pressure the hydrogel membrane. After 15 days, it was found that a small amount of liquid penetrated into the originally empty right vessel, indicating excellent water-storing capacity and permeability of the hydrogel membrane. It is worth noting that by adding some pore-forming materials, such as CaCO_3_ and Na_2_CO_3_, or some surfactant during the synthetic process of hydrogel, macrogels and porous gels can be obtained with enhanced penetration of the hydrogel membrane.

### Adsorption and Photocatalytic Degradation Recycles

**Figure 5:**
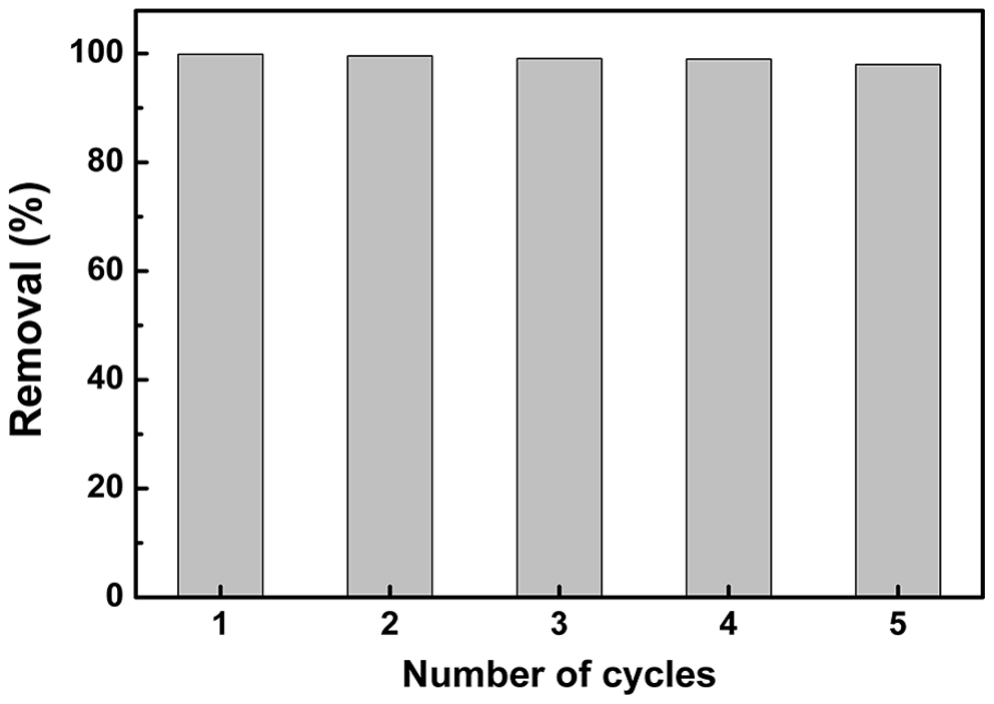
Cycle and reuse of the hydrogel membrane.

The hydrogel membrane can effectively reduce the overall cost of adsorbent because of its high adsorption capacity, good separation selectivity and excellent reusing ability. In order to recycle the hydrogel membrane, several methods were tried, such as stripping MB with 0.1 M HCl, 0.5 M H_2_SO_4_, or photocatalytic degradation of MB by Fenton reagent. The results demonstrated that Fenton reagent was a suitable candidate for photocatalytic degradation of MB. In each adsorption–photodegradation cycle, nearly 100% loading and photodegradation can be realized on condition that the initial concentration of MB is 10.0 mg L^−1^. Such cycle can be repeated at least five times (Figure 5). Except that a small amount of Fe^3+^ remained in the hydrogel, no any other obvious decrease in mechanical properties could be observed.

In summary, a new and versatile application for tough hydrogel was successfully developed by constructing an elastic adsorption membrane, which can be used as a platform for removal and photocatalytic degradation of methylene blue. The as-prepared transparent clay nanocomposite hydrogel has not only excellent mechanical and swelling properties, but also high adsorption capacity, good separtion selectivity and repeated service life. Moreover, it is anticipated that this type of elastic and tough hydrogel membrane-based separation technique will be particularly suitable for the detoxification of industrial effluent and the recovery of valuables.

## Supporting Information

Figure S1 **Stress-strain curves of hydrogels in various compositions and status.** (A) Compressive properties. (B) Tensile properties.
(TIF)

Figure S2 **The adsorption of crystal violet and acid fuchsin with clay nanocomposite hydrogel.** (RT, pH of 6.5 and 150 mL)
(TIF)

Figure S3 **The adsorption of Cu^2+^ and Pb^2+^ with clay nonacomposite hydrogel.** The initial concentrations of metal ions are both 20.0 mg L^−1^ (RT, 150 mL).
(TIF)

Table S1 **Swelling ratio of hydrogels in various concentrations and different status.**
(DOC)

Table S2 **Mechanical properties of hydrogels in various compositions and status.**
(DOC)
