# Supplementary figures and images for: Functional Elastic Hydrogel as Recyclable Membrane for the Adsorption and Degradation of Methylene Blue
Source: PLoS One. 2014 Feb 20;9(2):e88802. doi: 10.1371/journal.pone.0088802 (PMC3930577; doi:10.1371/journal.pone.0088802)

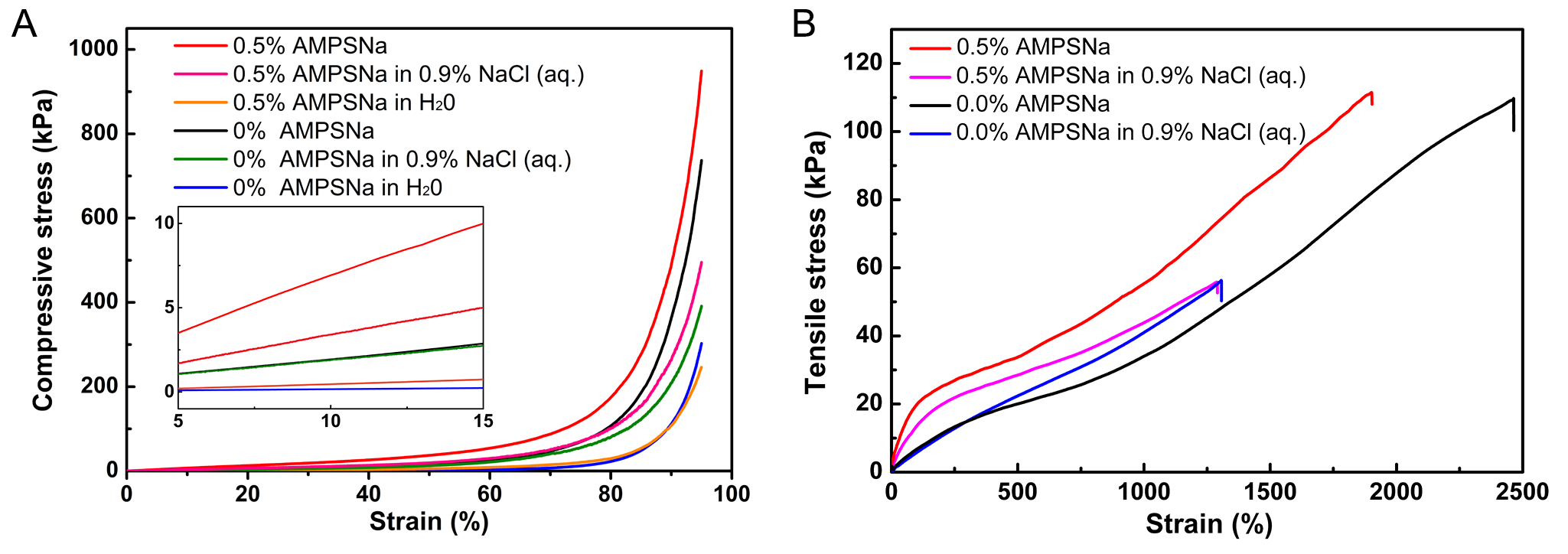

Supplement: Figure S1 — Stress-strain curves of hydrogels in various compositions and status. (A) Compressive properties. (B) Tensile properties. (TIF) [file pone.0088802.s001.tif]

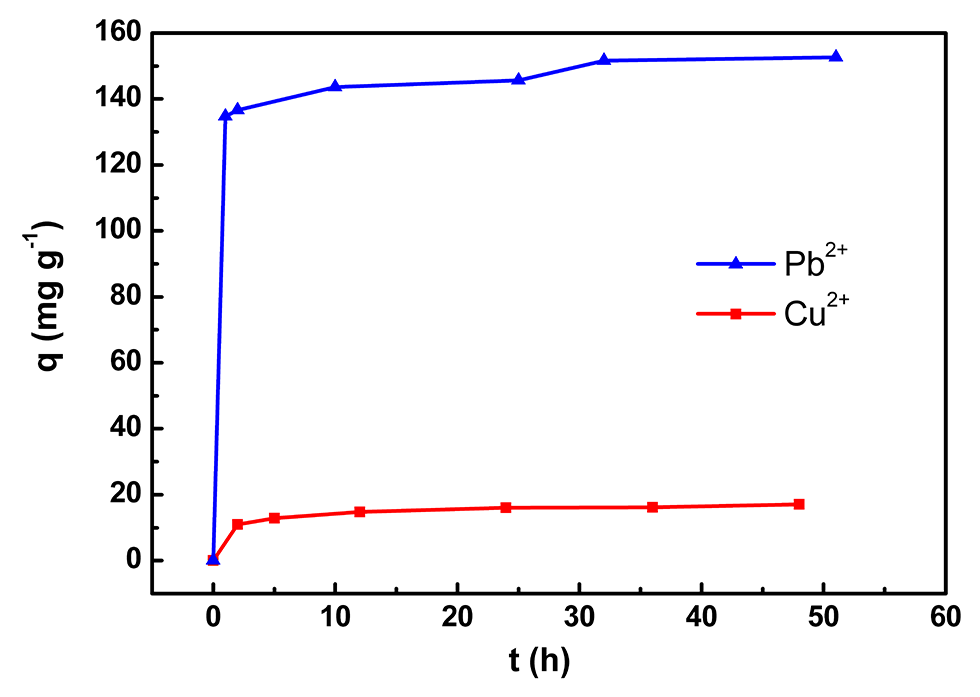

Supplement: Figure S3 — The adsorption of Cu2+ and Pb2+ with clay nonacomposite hydrogel. The initial concentrations of metal ions are both 20.0 mg L−1 (RT, 150 mL). (TIF) [file pone.0088802.s003.tif]
