# Supplementary material for: Functional Elastic Hydrogel as Recyclable Membrane for the Adsorption and Degradation of Methylene Blue
Source: PLoS One. 2014 Feb 20;9(2):e88802. doi: 10.1371/journal.pone.0088802 (PMC3930577; doi:10.1371/journal.pone.0088802)
Supplement: Table S1 — Swelling ratio of hydrogels in various concentrations and different status. (DOC) [file pone.0088802.s004.doc]

**Table S1. Swelling ratio of hydrogels in various concentrations and different status**

| **Compositions** | | **Swelling Ratio** | |
| --- | --- | --- | --- |
| **DMAA/AMPSNa (wt%/wt%)** | **Clay-NS (wt%)** | **in distilled water** | **in 0.9% NaCl solution** |
| 5.0/0.0 | 5.0 | 80.5 ± 1.9 | 13.4 ± 0.2 |
| 4.7/0.3 | 5.0 | 162.2 ± 3.2 | 14.4 ± 0.3 |
| 4.5/0.5 | 5.0 | 172.0 ± 3.4 | 14.5 ± 0.3 |
| 4.3/0.7 | 5.0 | 225.1 ± 5.2 | 21.8 ± 0.4 |
