# Supplementary material for: Functional Elastic Hydrogel as Recyclable Membrane for the Adsorption and Degradation of Methylene Blue
Source: PLoS One. 2014 Feb 20;9(2):e88802. doi: 10.1371/journal.pone.0088802 (PMC3930577; doi:10.1371/journal.pone.0088802)
Supplement: Table S2 — Mechanical properties of hydrogels in various compositions and status. (DOC) [file pone.0088802.s005.doc]

**Table S2.** **Mechanical properties of hydrogels in various compositions and status**

| **No.** | **DMAA/AMPSNa (****wt%/wt%)** | **Comp*. modulus (kPa)** | **Comp*. strength (kPa)** | **Tensile modulus (kPa)** | **Tensile strength (kPa)** | **Elongation (%)** |
| --- | --- | --- | --- | --- | --- | --- |
| 1 | 5.0/0.0 ^a^ | 17.4 ± 0.3 | 736.9 ± 21.0 | 9.3 ± 0.3 | 109.7 ± 6.6 | 2464 ± 89 |
|  | 5.0/0.0^b^ | 16.7 ± 0.3 | 391.3± 15.3 | 9.1 ± 0.3 | 56.5 ± 4.1 | 1305 ± 61 |
|  | 5.0/0.0 ^c^ | 1.3 ±0.1 | 302.8± 29.7 |  |  |  |
| 2 | 4.8/0.2 ^a^ | 38.5 ± 1.8 | 810.0 ± 30.2 |  |  |  |
| 3 | 4.6/0.4 ^a^ | 45.9 ± 2.3 | 900.1 ± 28.8 |  |  |  |
| 4 | 4.5/0.5^a^ | 64.9 ± 2.4 | 949.2 ± 27.5 | 29.4 ± 1.2 | 111.8 ± 7.0 | 1901 ± 92 |
|  | 4.5/0.5^b^ | 33.0 ± 1.8 | 495.4 ± 23.5 | 22.3 ± 1.1 | 56.0 ± 3.9 | 1286 ± 58 |
|  | 4.5/05 ^c^ | 5.2 ± 0.5 | 246.2 ± 25.1 |  |  |  |
| 5 | 4.4/0.6 ^a^ | 50.5 ± 2.9 | 888.9 ± 33.7 |  |  |  |
| 6 | 4.1/0.9 ^a^ | 46.7 ± 2.6 | 816.8 ± 31.9 |  |  |  |

a = fresh hydrogel

b = hydrogel swelled in 0.9% NaCl solution

c = hydrogel swelled in distilled water

Comp.* = Compressive

Comp.* strength: at a strain of 95%
